# Strong association between the dietary inflammatory index(DII) and breast cancer: a systematic review and meta-analysis

**DOI:** 10.18632/aging.202985

**Published:** 2021-05-07

**Authors:** Huajian Chen, Yuzhe Gao, Na Wei, Kuiying Du, Qi Jia

**Affiliations:** 1 Department of Breast Surgery, Guizhou Provincial People’s Hospital, Guizhou, China

**Keywords:** dietary inflammatory index, breast cancer

## Abstract

The association between the Dietary Inflammatory Index (DII) and breast cancer risk has been widely reported in recent years, but there is still controversy about whether a pro-inflammatory diet is a risk factor for breast cancer. We conducted a meta-analysis to investigate the relationship between the DII and breast cancer risk in pre-menopausal and post-menopausal women. We comprehensively searched PubMed, Embase and the Cochrane Library in January 2021 to identify articles reporting an association between the DII and breast cancer risk. A pooled analysis was conducted with 14 studies covering 312,885 participants. Overall, women in the most pro-inflammatory diet category were at greater risk for breast cancer than those in the most anti-inflammatory category (relative risk [RR]=1.37, 95% confidence interval [CI] 1.17-1.60, P<0.001). This association was strong in both pre-menopausal women (RR=1.87, 95% CI 1.17-2.99, P=0.001) and post-menopausal women (RR=1.23, 95% CI 1.08-1.40, P<0.001). Thus, a strong and independent association was observed between a pro-inflammatory diet (assessed using the DII score) and breast cancer risk, irrespective of menopausal status. Further studies will be required to determine the relationship between a pro-inflammatory diet and different subtypes of breast cancer.

## INTRODUCTION

Breast cancer has become the most common cancer among women, and the number of new cases has been increasing rapidly [1]. Other than reproductive, hormonal and genetic factors, inflammation has been reported as a potential cause of breast cancer [2]. The production of pro-inflammatory cytokines and diverse reactive oxygen and nitrogen species during inflammation facilitates cancer initiation, growth and malignant progression [3].

In addition to autoimmune diseases and infections, several risk factors related to lifestyle may lead to chronic inflammation. One such risk factor is the dietary composition. Saturated fats, refined carbohydrates and red meat may exert pro-inflammatory effects, while soy products and phytochemicals may exert anti-inflammatory effects due to their influence on oxidative stress and other pathways [4].

The Dietary Inflammatory Index (DII) was created to comprehensively investigate the inflammatory potential of the diet in the general population with adjustment for dietary differences around the world. This index was developed through an extensive review of over 6500 articles published from 1950 to 2010. Forty-five food parameters (including various macronutrients, micronutrients, flavonoids and individual food items) were evaluated, and six inflammatory biomarkers were used to calculate the inflammatory characteristics of each food parameter. A food parameter was considered pro-inflammatory if its intake was associated with significant increases in inflammatory biomarkers, or anti-inflammatory if it was associated with significant decreases in inflammatory biomarkers. A score was assigned to each food parameter based on its association with inflammatory biomarkers in the literature, and the score was weighted based on study design. The scores were then adjusted to the global standard mean intake, converted into proportions (0-1) and distributed around zero, with higher DII scores indicating pro-inflammatory effects and lower DII scores suggesting anti-inflammatory effects [5].

The relationship between the DII and breast cancer risk has been widely studied in recent years, but the results have been inconclusive. Shivappa et al. [6] reported that diets with high DII scores appeared to increase the risk of breast cancer, while Gardeazabal et al. [7] reported no association between the DII and breast cancer risk. Other researchers also have not found a significant association between the DII and breast cancer risk, but indicated that further studies with longer follow-up periods were needed to clarify the association [8, 9]. In addition, the association between the DII and breast cancer risk in different populations and breast cancer subtypes has not been fully investigated. Therefore, in this study, we conducted a systematic review and meta-analysis to explore the association between the DII and breast cancer risk using a large sample size, and to further assess this relationship in different populations and breast cancer subtypes.

## RESULTS

### Study characteristics

**Figure 1:**
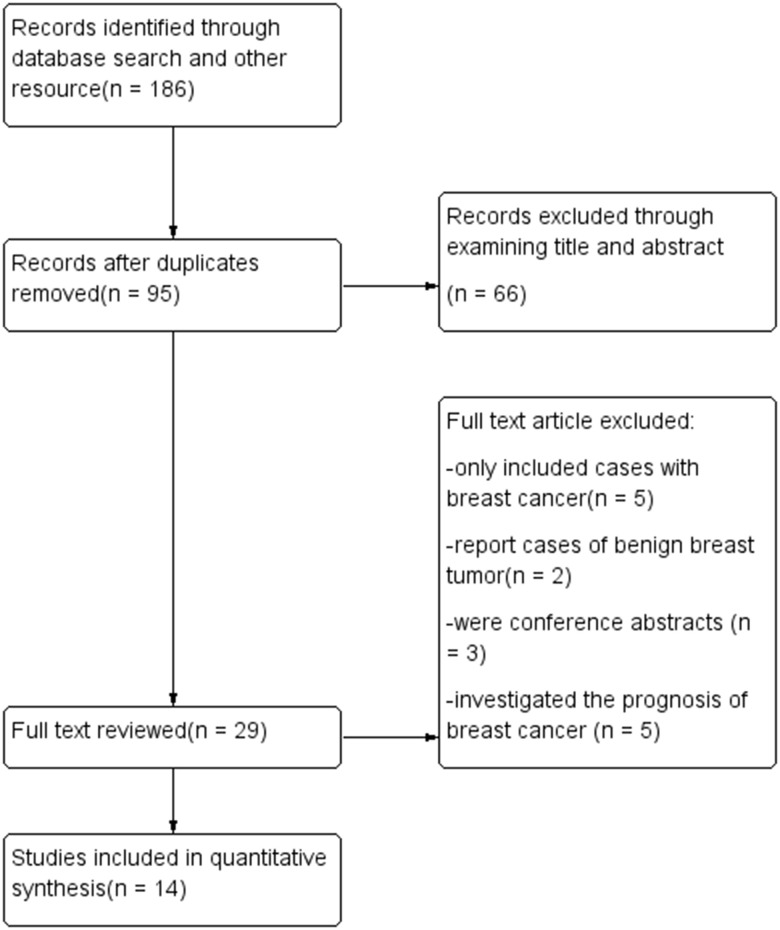
Flow diagram.

Fourteen studies were included in this meta-analysis (Figure 1), of which six were cohort studies and eight were case-control studies. In total, 312,885 participants were included in the pooled analysis. The main characteristics of the included studies are listed in Table 1. The DII was determined based on food frequency questionnaires in all the included studies, with the number of food parameters ranging from 22 to 37. The DII was calculated and adjusted using a regionally representative world dataset. Multivariate adjustment was applied based on age, body mass index, education, energy intake, family cancer history, hormone use, live birth number, menopausal status, menarche age and smoking status. Odds ratios (ORs) and relative risks (RRs) were determined between the most pro-inflammatory and most anti-inflammatory groups. In the cohort studies, the follow-up periods ranged from 10.3 to 25 years.

**Table 1:** Characteristics of the included studies.

**Author and year**	**Country**	**Case no.**	**Control no.**	**Source of controls**	**Food parameters**	**NOS score**	**OR/RR with 95% CI**
**Case-Control**							
Ge 2015	Germany	2887	5512	Community	25	7	1.01 (0.86-1.17)
Huang 2016	China	867	824	Hospital	36	8	2.28 (1.71-3.03)
Shivappa 2017	Italy	2569	2588	Community	31	8	1.75 (1.39-2.21)
Jalali 2018	Iran	136	272	Hospital	34	6	1.30 (1.56-10.08)
Vahid 2018	Iran	145	148	Hospital	31	7	7.24 (3.14-16.68)
Lee 2019	Korea	364	364	Hospital	37	7	3.62 (2.34-5.80)
Santacana 2019	Spain	1486	1652	Community	30	7	1.22 (0.99-1.52)
Niclis 2020	Argentina	317	526	Community	22	6	1.34 (1.05-1.70)
**Cohort**		**Case no.**	**Cohort no.**	**FU (yrs)**			
Shivappa 2015	Swedish	1895	49,258	20	29	8	1.18 (1.00-1.39)
Tabung 2016	USA	8162	122,788	15.1	32	8	0.99 (0.91-1.07)
Graffoullere 2016	France	158	3771	12.6	36	8	0.85 (0.52-1.41)
Tabung 2017	USA	3471	70,998	16.05	32	8	1.03 (0.90-1.17)
Shivappa 2018	USA	1986	34,700	25	29	8	1.11 (1.00-1.22)
Gardeazabal 2018	Spain	100	10,713	10.3	28	8	1.44 (0.72-2.72)

NOS: Newcastle-Ottawa Scale, FU: Follow-up.

### Quantitative analysis

**Figure 2:**
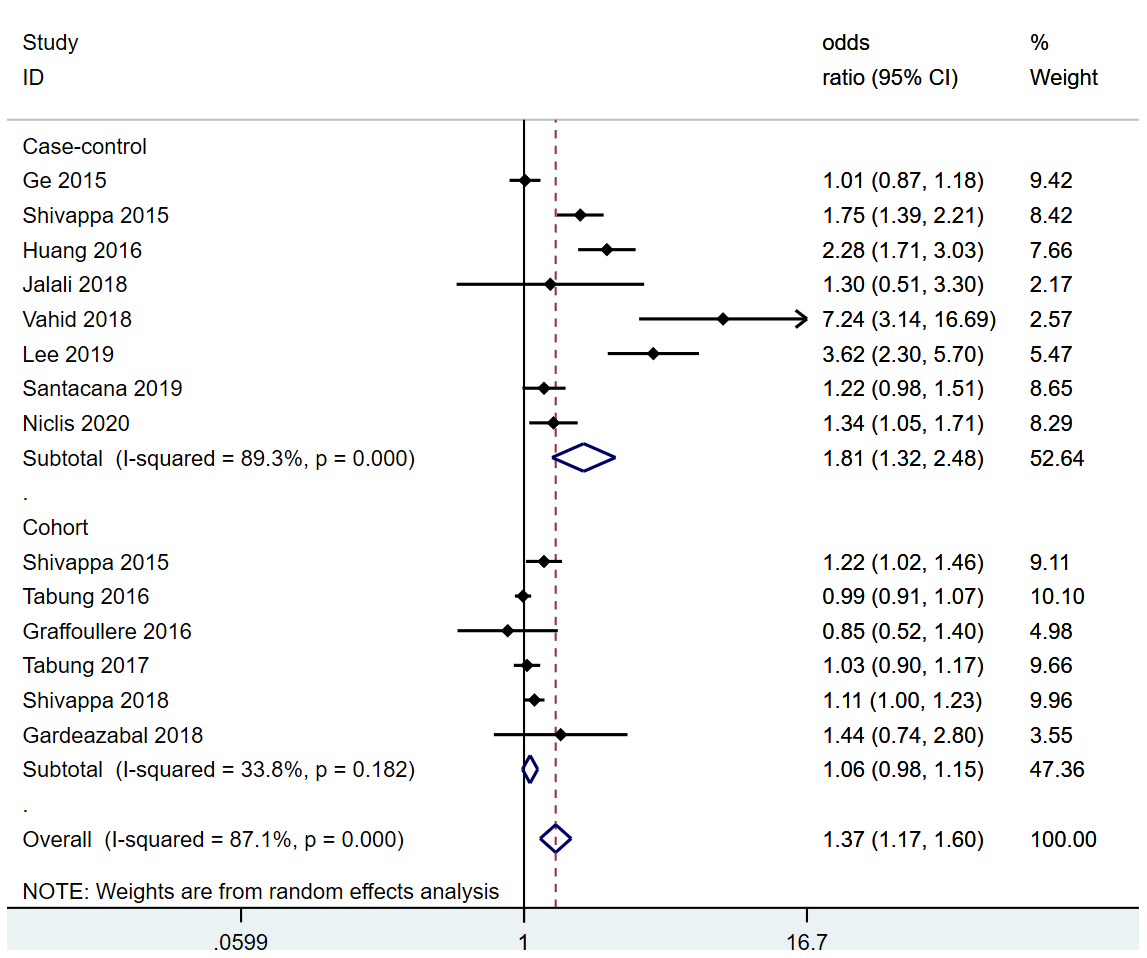
Pooled analysis of association between DII and breast cancer.

Overall, women in the most pro-inflammatory diet category were at significantly greater risk for breast cancer than those in the most anti-inflammatory category (RR=1.37, 95% confidence interval [CI] 1.17-1.60, P<0.001) (Figure 2). This significant increase in risk was also observed when the pooled analysis was restricted to case-control studies (RR=1.81, 95% CI 1.32-2.48, P<0.001), whereas a moderate increase in risk was observed when the analysis was restricted to cohort studies (RR=1.06, 95% CI 0.98-1.15, P=0.182).

Next, a subgroup analysis was conducted based on menopausal status. In post-menopausal women, a pooled analysis including 11 studies demonstrated that the risk of breast cancer in the most pro-inflammatory diet group was significantly greater than that in the most anti-inflammatory group (RR=1.23, 95% CI 1.08-1.40, P<0.001). Likewise, in pre-menopausal women, a pooled analysis including six studies demonstrated that the risk of breast cancer in the most pro-inflammatory diet group was significantly greater than that in the most anti-inflammatory group (RR=1.87, 95% CI 1.17-2.99, P=0.001).

Seven pro-inflammatory dietary components were included in this meta-analysis: vitamin B12, carbohydrates, cholesterol, total fat, protein, saturated fat and trans-fat. Carbohydrate, cholesterol and total fat intake were assessed by all 14 studies, whereas vitamin B12 and saturated fat intake were assessed by 13 studies and protein intake was assessed by 12 studies. Subgroup analyses were conducted on studies that included particular food components. No significant difference in breast cancer risk was observed based on the inclusion of any specific food component in the DII. However, the breast cancer risk associated with the most pro-inflammatory diet was moderately higher in the subgroup analyses of studies that included vitamin B12 (RR=1.42, 95% CI 1.19-1.71) or saturated fat in the DII (RR=1.40, 95% CI 1.19-1.64) than in the subgroup analyses of studies that included the other specific components (Table 2).

**Table 2:** Subgroup analysis of studies that assessed different food components.

**Component**	**Inflammatory score based on the DII^5^**	**Number of studies**	Risk of breast cancer*
Vitamin B12 (ug)	0.106	13	1.42 (1.19-1.71)
Carbohydrates (g)	0.097	14	1.37 (1.17-1.60)
Cholesterol (mg)	0.11	14	1.37 (1.17-1.60)
Total fat (g)	0.298	14	1.37 (1.17-1.60)
Protein (g)	0.021	12	1.38 (1.16-1.63)
Saturated fat (g)	0.373	13	1.40 (1.19-1.64)
Trans-fat (g)	0.229	5	1.30 (1.05-1.60)

*A subgroup analysis was conducted on the association between the DII and breast cancer risk in studies that included the specified food component in the DII. The results are shown as the RR with 95% CI.

### Sensitivity analysis

**Figure 3:**
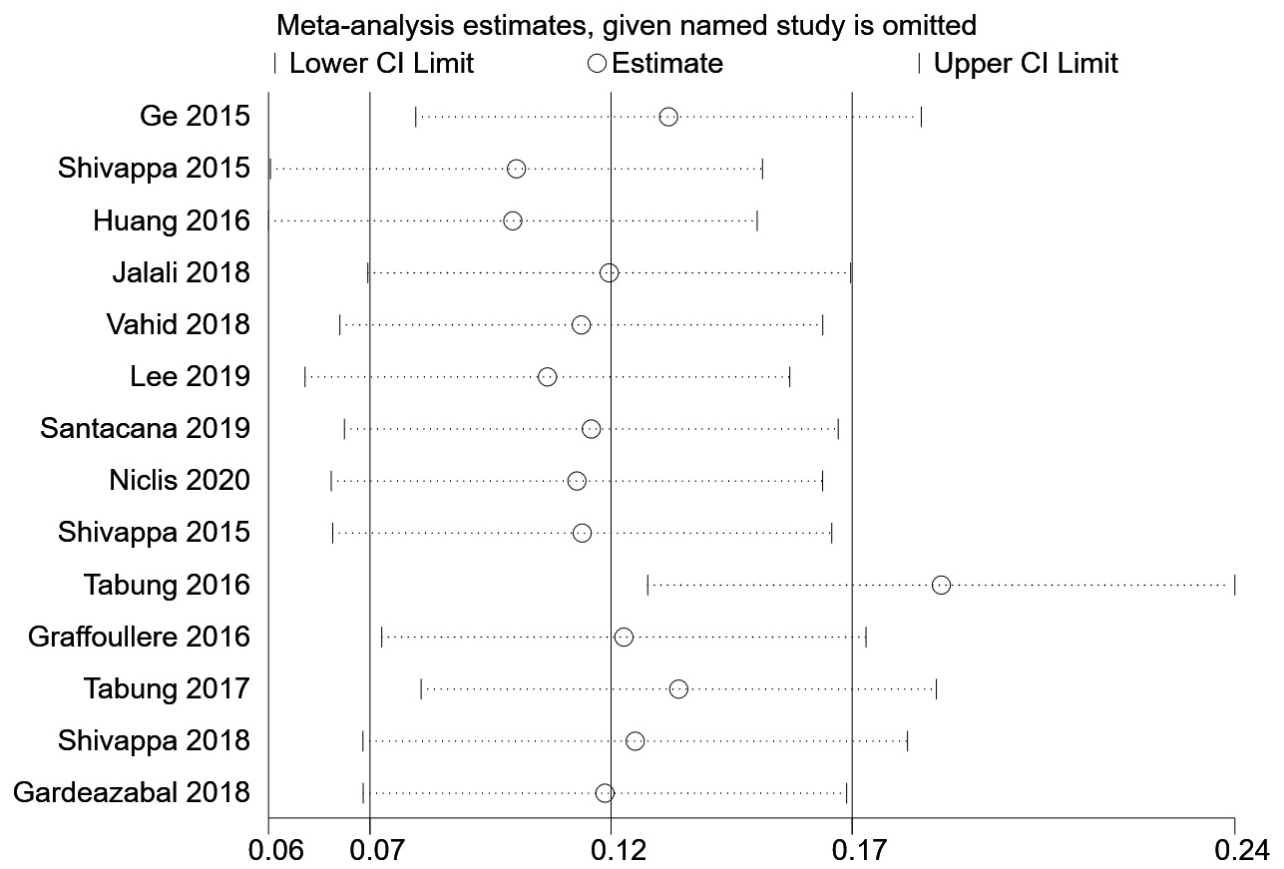
Galbraith plot.

**Figure 4:**
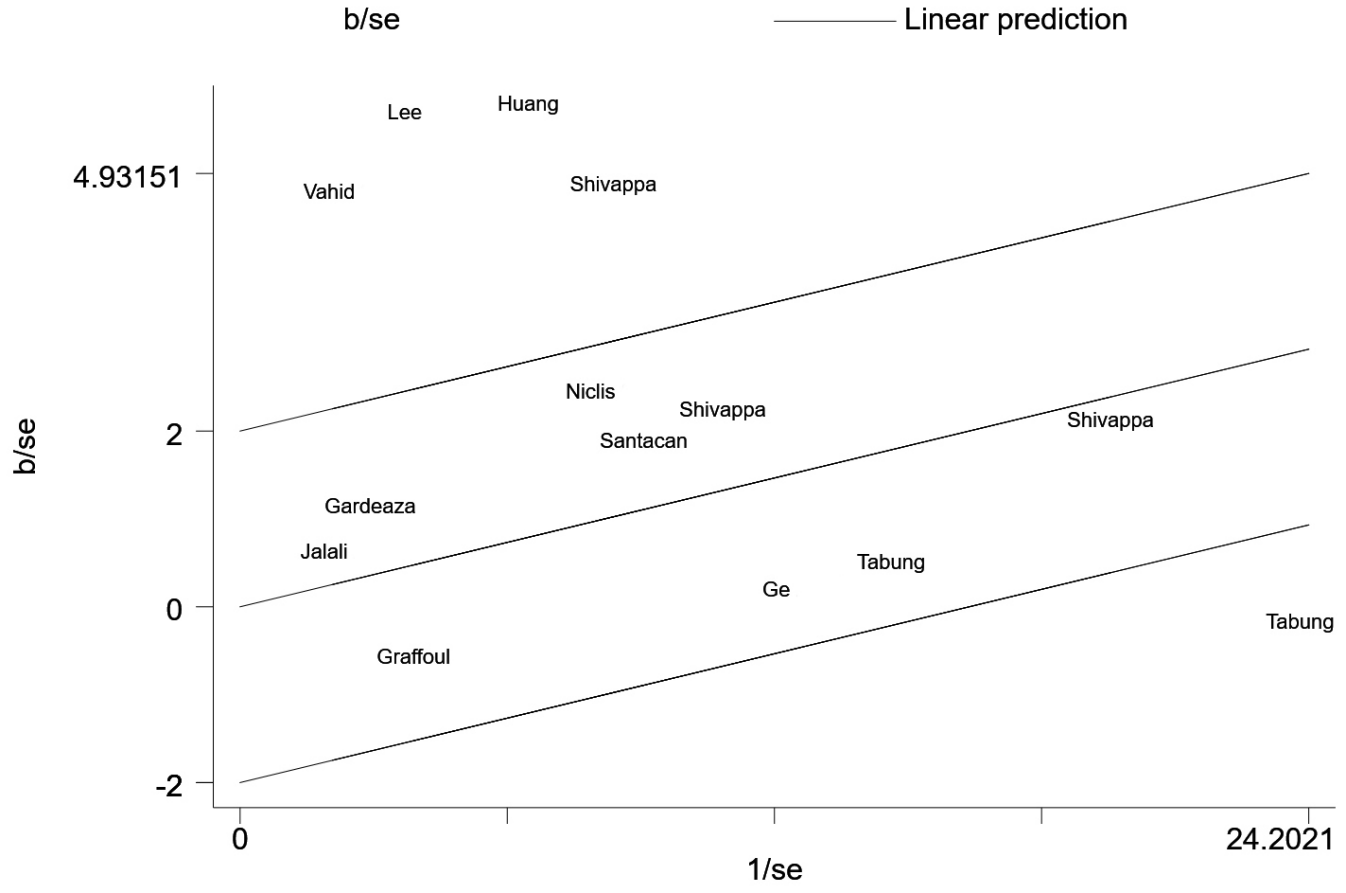
Influence analysis.

We then conducted a heterogeneity analysis, which revealed substantial heterogeneity among the included studies (I^2^=87.1%). A Galbraith plot (Figure 3) demonstrated that five studies were outside the 95% CI. A sensitivity analysis excluding these five studies was conducted, and the results indicated that the risk of breast cancer in the most pro-inflammatory diet group was still greater than that in the most anti-inflammatory group (RR=1.11, 95% CI 1.04-1.17, P=0.424). To further address the heterogeneity among the studies, we conducted an influence analysis in which each study was removed in turn (Figure 4). One study seemed to be the major cause of heterogeneity, and a sensitivity analysis excluding this study still revealed a strong association between the DII and breast cancer risk (RR=1.43, 95% CI 1.20-1.70, P<0.001).

## DISCUSSION

Chronic inflammation has been linked to various malignancies, including breast cancer [10]. Inflammatory cytokines are secreted during chronic inflammation, and numerous studies have indicated that serum interleukin (IL)-6 levels are greater in breast cancer patients than in healthy controls. Cytokines such as IL-1β and tumor necrosis factor α have also been linked to the risk and prognosis of breast cancer [11–13].

Clinical studies have indicated that pro-inflammatory dietary components may cause chronic inflammation and elevate inflammatory biomarker levels. A small number of *in vitro* studies have also investigated the association between food parameters and inflammation; for instance, Müller et al. [14] found that oleic acid exerted anti-inflammatory effects by attenuating inducible nitric oxide synthase, cyclooxygenase 2, tumor necrosis factor α, IL-1β and IL-6 mRNA expression in lipopolysaccharide-treated macrophages. However, there is no direct link between DII and breast cancers can be found in experimental data. Carbohydrates, cholesterol and saturated fat have been considered as pro-inflammatory food components, while pepper, caffeine, onions, green tea and garlic have been considered as anti-inflammatory. Nevertheless, people in different regions of the world vary greatly in their dietary habits and food parameters, making it difficult to assess the overall inflammatory effects of individual diets.

In order to systematically investigate the inflammatory effects of the diet, Shivappa et al. [5] developed the DII, in which each participant is standardized to the world mean and standard deviation for his/her respective food parameters. Therefore, participants from different regions of the world are standardized in each study using the DII as an assessment tool. In this meta-analysis, we evaluated the association between the DII and breast cancer risk. Seven pro-inflammatory dietary components were included: vitamin B12, carbohydrates, cholesterol, total fat, protein, saturated fat and trans-fat.

The primary finding of this study was that a pro-inflammatory diet was an independent risk factor for breast cancer after adjustment for covariates including age, body mass index, energy intake, education, menopausal status and live birth number. In some studies, additional adjustments were applied for mammography, non-steroidal anti-inflammatory drug use and oophorectomy. A subgroup analysis was conducted according to menopausal status, and a significant increase in breast cancer risk for the most pro-inflammatory diet group was found in both pre-menopausal and post-menopausal women. Major pro-inflammatory dietary components were assessed in most studies, and subgroup analyses revealed that the association between the DII and breast cancer risk was stronger in studies that included vitamin B12 and saturated fat as pro-inflammatory dietary components.

Cohort studies and case-control studies were both included in this meta-analysis [7, 15–23]. Case-control studies are more prone to bias due to their retrospective nature. The majority of the included studies reported moderate to significant associations between the DII and breast cancer risk, although two of the cohort studies (Tabung et al. [16] and Gardeazabal et al. [7]) reported no association. Accordingly, a subgroup analysis that only included the cohort studies revealed a more moderate association between the DII and breast cancer risk. Aside from the studies included in this meta-analysis, one previous study found no association between the DII and breast cancer-specific deaths [24], while Aghababayan et al. [25] reported a possible association between the DII and benign breast tumors.

A previous meta-analysis that included seven observational studies concluded that there was no significant association between the DII and breast cancer risk [8]. Our meta-analysis included 14 studies, and revealed that the most pro-inflammatory DII scores were associated with a significant increase in breast cancer risk after adjustment for covariates. This difference may have been due to the increased sample size and number of studies included in our analysis.

This study had several limitations. Due to insufficient data, we were unable to analyze the association between the DII and different subtypes of breast cancer based on hormone receptor expression and pathological characteristics. Cohort studies and case-control studies were both included in this meta-analysis, which could have caused bias; however, a subgroup analysis was conducted based on study design. Substantial heterogeneity was found among the included studies, but sensitivity and influence analyses yielded similar results to our primary findings. The strengths of this study were that the incidence of breast cancer was low, that a pooled analysis with a large sample size was conducted using the existing evidence, and that adjustments were made for other breast cancer risk factors.

In conclusion, a strong and independent association was observed between a pro-inflammatory diet (assessed using the DII score) and breast cancer risk. This association was strong in both pre-menopausal and post-menopausal women. Further studies are required to investigate the association between pro-inflammatory diets and different subtypes of breast cancer.

## MATERIALS AND METHODS

### Data source and search strategy

PubMed, Embase and the Cochrane Library were comprehensively searched in January 2021. The search terms included: Dietary inflammatory index, DII and breast cancer. The search terms used in PubMed were (((“diet”[MeSH Terms] OR “diet”[All Fields] OR “dietary”[All Fields] OR “dietaries”[All Fields]) AND (“inflammation”[MeSH Terms] OR “inflammation”[All Fields] OR “inflammations”[All Fields] OR “inflammation s”[All Fields]) AND (“abstracting and indexing”[MeSH Terms] OR (“abstracting”[All Fields] AND “indexing”[All Fields]) OR “abstracting and indexing”[All Fields] OR “index”[All Fields] OR “indexed”[All Fields] OR “indexes”[All Fields] OR “indexing”[All Fields] OR “indexation”[All Fields] OR “indexations”[All Fields] OR “indexe”[All Fields] OR “indexer”[All Fields] OR “indexers”[All Fields] OR “indexs”[All Fields])) OR “DII”[All Fields]) AND (“breast neoplasms”[MeSH Terms] OR (“breast”[All Fields] AND “neoplasms”[All Fields]) OR “breast neoplasms”[All Fields] OR (“breast”[All Fields] AND “cancer”[All Fields]) OR “breast cancer”[All Fields]). All articles published before January 1, 2021 were screened. Additional studies were identified from the retrieved articles.

### Inclusion and exclusion criteria

The titles and abstracts of the retrieved articles were screened for relevant studies, and the full texts of studies of interest were screened by two independent researchers. Clinical trials were included if they: 1) assessed the relationship between the DII and the risk of breast cancer in humans and 2) reported quantitative data. Studies were excluded if they: 1) only included patients who had already been diagnosed with breast cancer; 2) investigated the prognosis of breast cancer; 3) did not report quantitative data or 4) were conference abstracts, animal studies, cadaveric studies, *in vitro* studies or articles published in a form other than a clinical trial.

### Data extraction and quality assessment

Two researchers extracted the data and assessed the quality of the included studies independently. The corresponding author was sought to resolve any disagreement. Data including the sample size, study design, population characteristics, RR, OR, 95% CI and other relevant details were recorded and verified. The Newcastle-Ottawa Scale was used to assess the quality of the included studies.

### Statistical analysis

Statistical analyses were performed using Stata 16 (StataCorp, College Station, TX, USA). Multivariable adjusted RRs were pooled to compare the most pro-inflammatory and most anti-inflammatory diets using a random effects model due to high heterogeneity. Because the incidence of breast cancer was low, the ORs reported in case-control studies were considered equal to RRs, in accordance with the Cochrane Handbook for Systematic Reviews. Subgroup analyses were conducted when no less than four studies could be included. Heterogeneity was assessed with I^2^ and a Galbraith plot. To address the high between-study heterogeneity, an influence analysis was conducted in which each study was removed in turn to examine the results of the pooled analysis.

### Ethical review committee statement

Since our study is a meta-analysis, an Ethical Review Committee Statement is not required.

The analysis was mainly conducted in Guizhou Provincial People’s Hospital.
